# Factors affecting work ability index among polish nurses working in hospitals – A prospective observational survey

**DOI:** 10.1111/jonm.13192

**Published:** 2020-11-16

**Authors:** Łukasz Rypicz, Izabela Witczak, Joanna Rosińczuk, Piotr Karniej, Anna Kołcz

**Affiliations:** ^1^ Department of Health Care Economics and Quality Wroclaw Medical University Wroclaw Poland; ^2^ Department of Nervous System Diseases Wroclaw Medical University Wroclaw Poland; ^3^ Department of Organisation and Management Wroclaw Medical University Wroclaw Poland; ^4^ Laboratory of Ergonomics and Biomedical Monitoring Wroclaw Medical University Wroclaw Poland

**Keywords:** nurse practitioners, occupational health, public health, quality of care, workplace ergonomics

## Abstract

**Aims:**

To assess the ability to work of Polish nurses by age groups.

**Background:**

The ability to work is widely discussed in the literature in the context of nurses' productivity; thus, it is necessary to identify the ability to work when facing an increasing demand for services.

**Methods:**

The observational study involved 349 professionally active nurses aged 46.9 ± 9.7 years, with a length of service of 23.5 ± 9.6 years. The Work Ability Index (WAI) was used to assess the nurses' ability to work.

**Results:**

The ability to work decreases with age (*r*_s_ = −0.324, *p* < .000) and with seniority (*r*_s_ = −0.257; *p* < .000). Nurses with higher education presented higher Work Ability Index scores. Also, the age (*B* = −0.25, *p* < .001), work seniority (*B* = −0.19, *p* < .001) and education (masters' degree: *B* = 1.41, *p* = .012; ref. secondary) affect work ability.

**Conclusions:**

The ageing process and seniority of nurses negatively affect their ability to work. A lack of programmes to maintain physical condition for nurses can result in a shortage of staff.

**Implications for nursing management:**

Programmes can be developed to create or improve healthy working environments to increase productivity.

## 1 BACKGROUND

Nurses far outnumber doctors in most countries of the Organisation for Economic Co‐operation and Development (OECD) and play a key role in providing health care not only in traditional settings such as hospitals and long‐term care institutions but also increasingly in primary care (especially chronic care) and home care services. Concerns about possible future shortages of nurses are growing in many OECD countries as the demand for nursing services is expected to rise when facing an ageing population and the gradual retirement of nurses belonging to the Baby Boom generation. These concerns have led many countries to increase the number of institutions for nurse training (Marć et al., 2019; Organisation for Economic Co‐operation & Development, 2017; Sherman et al., 2013).

An increasing number of patients requiring nursing care without an increase in the number of actively employed nurses may result in a severe deterioration in the quality of services, and consequently an increased risk of adverse events and higher mortality rates (Kruk et al., 2018). It was noted that each additional patient per nurse was associated with a 7% higher probability of death within 30 days of admission and a 7% higher chance of failure to rescue (Aiken et al., 2002).

In Poland, the shortage of nursing staff is caused, among other things, by low wages and emigration to countries with higher earnings (Marć et al., 2019). The fact is that the migration of the new generation of Polish nurses should now become a significant component of human resources policy in the Polish health service (Szpakowski et al., 2019).

It should be emphasized that one of the indicators accessing nursing care is the ratio of nursing personnel per 1,000 inhabitants. In Poland, this indicator's value is 5.24, which is very low compared to Switzerland, Denmark or Germany, 17.56, 15.52 and 13.14, respectively (Abbasi et al., 2017). With the current dynamics of the population ageing, it is recommended that policies be introduced to raise the value to the average of OECD countries, which is 9.4 nurses per 1,000 inhabitants (Marć et al., 2019; Polish Chamber of Nurses & Midwives, 2017a, 2017b; Proniewicz, 2017).

Work ability is a fundamental social issue because it affects workers' health and well‐being (Mehrdad et al., 2016). Besides, the ability to work in a subjective assessment made by the employee results from the requirements of his work and the psychophysical abilities, which are personal characteristics.

Employers, more and more often, pay attention to the ability of their employees to work. With demographic changes increasing the proportion of older people in the population, employability is becoming crucial. Demographic projections in 27 European countries assume that over four decades – from 2010 to 2050 – the ratio of the post‐working‐age population to the working‐age population (15–64) will double from 25.9% to 50.2% (European Commission, 2015).

Given the ageing population, which is becoming a global challenge for the health care sector, the increase in demand for nursing services will be closely linked to nurses' availability in the labour market. This means that nurses' physical and mental health should be at the highest possible level, translating into a high capacity to work. Nurses' ability to work is one of the topics discussed in the literature in the context of productivity when performing professional duties (Heyam et al., 2018).

It was decided to conduct a survey study among Polish nurses on the ability to work mainly due to the high workload (the result of the shortage of nurses in the labour market) and the ageing process. It was investigated how the ability to work is affected by age, work seniority and nurses' level of education. The literature review shows that the ability to work may vary according to age group (Hatch et al., 2018) or education level (Golubic et al., 2009).

This study points out the decreasing ability to work among Polish nurses and indicates the need for multidimensional actions to prevent the total paralysis of nursing care, which can be faced by many European countries with a dynamically ageing population. Therefore, the study aimed to assess the ability to work in a professionally active group of nurses by age group. It has been hypothesized that the index of work ability decreases with nurses' age.

## 2 METHODS

### 2.1 Design and settings

The observational study was carried out from July 2018 to August 2018 in four Wroclaw hospitals: (a) Provincial Specialist Hospital, (b) Provincial Specialist Hospital, Research and Development Center, (c) University Clinical Hospital and (d) Military Clinical Hospital with Polyclinic. The STrengthening the Reporting of OBservational studies in Epidemiology (STROBE) guidelines for reporting of observational studies were followed (von Elm et al., 2007).

### 2.2 Study participants

The criterion for inclusion in the study was the active employment of the respondents as a registered nurse. The study initially included 360 nurses, 11 of whom were excluded because they failed to complete the questionnaire. Finally, the study group consisted of nurses working in surgical and conservative wards. The size of the study group was 349 participants (347 female and 2 male).

### 2.3 Data collection

The questionnaires were distributed to the nurses during their briefing about the research in the above‐mentioned hospitals' conservative and surgical wards at the beginning of July 2018. Participation was voluntary and anonymous. After completing the questionnaire, each nurse placed it in an envelope and turned it in at the agreed location. At the end of August 2018, the questionnaires were collected and statistically analysed.

### 2.4 Research tool

Data were collected using the Work Ability Index (WAI) questionnaire, which is used to assess health care professionals' level of work ability. The questionnaire was adapted for use in Poland by Pokorski (1998). Through a self‐assessment questionnaire, the WAI measures a worker's work ability and helps define necessary measures for maintaining and promoting work ability, as it helps to detect work‐related health risks as early as possible. Appropriate action can then be taken, including occupational health care, to prevent declining capacity and early retirement.

The WAI results from research by the Finnish Institution of Occupational Health (FIOH) employees (Tuomi et al., 1998). Their work aimed to develop a tool that could be used in occupational medicine to maintain work ability. The WAI expresses the subjective assessment of the worker's fitness for work. This tool was used by FIOH many times. In longitudinal studies, WAI allowed predicting changes in the ability to work in different professional groups. Also, the WAI provides a basis for future corrective and preventive action to improve or maintain employability.

The WAI questionnaire includes the following dimensions of the individuals: their current ability to work in comparison with their best years of life; their ability to work concerning their demand for work; the number of diagnosed diseases or limitations from which they suffer; their estimated impairments due to diseases/abilities or limitations; the number of sick leaves they have taken during the last year; the projections of their ability to work for 2 years (Juszczyk et al., 2019). Previous studies confirmed the validity and reliability of the WAI tool (Abbasi et al., 2016; Abdolalizadeh et al., 2012; Ebener & Hasselhorn, 2019; Peralta et al., 2012).

The WAI score ranges from 7 to 49 points and determines the employee's perception of work ability. The point value allows classifying the level of individual ability to work into the appropriate category and specifies the required preventive and intervention measures' objectives. A score of 7–27 points corresponds to low work ability ‐ restore work ability; a score of 28–36 points corresponds to moderate work ability ‐ improve work ability; a score of 37–43 points corresponds to good work ability ‐ support work ability; finally, a score of 44–49 points corresponds to excellent work ability ‐ maintain work ability (Adel et al., 2019).

### 2.5 Ethical considerations

The research project was approved by the independent Bioethics Committee of the Wroclaw Medical University (no. KB–240/2018 and KB–426/2018). All participants gave written informed consent after a thorough explanation of the procedures involved. The study was carried out following the tenets of the Declaration of Helsinki.

### 2.6 Statistical analysis

Sample size analysis was performed in Statistica 13 (TIBCO Software Inc., USA). Based on the results obtained in the hospital, the sample size was assessed in a pilot study. It was assessed how the ability to work in a professionally active group of nurses differed according to age. The pilot study was conducted among nurses aged under 30 and over 30. Means and standard deviations of WEI results in both groups were used to estimate the sample size. The sample size was estimated with a two‐sample unpaired‐means test (unpaired t test). Parameters: the mean score in the youngest group was 40.4 (SD = 6.1); the mean score in the oldest group was 38.1 (SD = 8.9); the alpha level was set at 0.05 and the power of the test at 0.9. It also assumed no correlation of evaluated variables and adopted a 2‐sided null hypothesis. The estimated sample size necessary was 316 participants based on the parameters. Also, the risk of losing participants (20%) was assumed. The final estimated sample size needed was 348 participants. The final sample size in this research was 349 participants.

The data obtained during the survey were collected and systematized using Microsoft Office Excel spreadsheet tools. The statistical analysis was conducted using Statistica 13 (TIBCO Software Inc., USA) under licence from the Wroclaw Medical University, Poland. For measurable variables, arithmetic means, medians, standard deviations, and the range of variability (extreme values) were calculated. For qualitative variables, the frequencies (per cent) were calculated. All investigated quantitative variables were checked with the Shapiro–Wilk test to establish the type of distribution. Comparative analysis was performed using non‐parametric tests – ANOVA Kruskal–Wallis rank with the post hoc test or Mann–Whitney *U* test. The correlation between the examined variables was performed using Spearman's rank correlation coefficient (*r*_s_)_._

An analysis of the impact of selected factors on work ability assessment was performed using linear regression (one‐factor model of predictors included in the analysis). A non‐standardized and standardized regression coefficient, standard error and level of statistical significance were determined. The next step was constructing the multi‐factor model (progressive step method), taking into account the variables whose *p*‐value in the univariate model was less than or equal to 0.50. For the overall study, a value of *p* < .05 was considered statistically significant.

## 3 RESULTS

### 3.1 Basic characteristics

The mean age of the studied nurses was 46.9 ± 9.7 years (median 48), and the mean length of service was 23.5 ± 9.6 years (median 26). Over 99% (*n* = 347) of the participants were female. Regarding age group distribution, the highest percentages were recorded in the age groups of 41–50 and 51–60 years: 42.7% and 34.4%, respectively (Table 1). The group characteristics also included education level, work seniority, Work Ability Index (WAI), work position, work in more than one place and hospital ward. Detailed information on the characteristics of the studied group is presented in Table 1.

**TABLE 1:** Characteristics of the studied group of nurses (*n* = 349)

Characteristic (variable)	Total (*n* = 349)
Age	
*M* ± *SD*	46.9 ± 9.7
Me (Q1; Q3)	48 (43; 53)
Min ÷ Max	20 ÷ 65
Work seniority	
*M* ± *SD*	23.5 ± 9.6
Me (Q1; Q3)	26 (19; 30)
Min ÷ Max	0.5 ÷ 44
Work Ability Index (WAI)	
*M* ± *SD*	34 ± 6.8
Me (Q1; Q3)	34 (29; 38)
Min ÷ Max	16 ÷ 49

	*N*	%
Sex		
Female	347	99.4
Male	2	0.6
Age group		
21–30	34	9.7
31–40	33	9.5
41–50	149	42.7
51–60	120	34.4
Over 60	13	3.7
Education		
Secondary	170	48.7
Bachelor's degree	91	26.1
Master's degree	88	25.2
Work position		
Ward nurse	29	8.3
Coordinating nurse	23	6.6
Unit nurse	263	75.4
Other position/e.g. surgical	34	9.7
Work in more than one place		
Yes	132	37.8
No	217	62.2
Hospital ward		
Conservative	187	53.6
Surgical	162	46.4

Abbreviations: *M*, mean; Max, maximum value; Me, median; Min, minimum value; *N*, number of participants; Q_1_, lower quartile; Q_3_, upper quartile; *SD*, standard deviation.

### 3.2 Comparison of results of WAI depending on selected variables

Statistically significant differences (*p* < .001) in the WAI score were found between ages 21–30 and ages 41–50, 51–60 and over 60, as well as between ages 31–40 and ages 41–50, 51–60 and over 60 (Table 2). The analysis of the WAI results showed a statistically significant correlation: the ability to work decreases with age (*r*_s_ = −0.32, *p* < .001). In the youngest age group (21–30), the WAI median's value was 42 points, which corresponds to the category of *good* ability to work. In the age group of over 60, the median WAI dropped to 31.5 points, which corresponds to the category of *moderate* work ability (Table 2).

**TABLE 2:** Comparison of results of WAI score

	The WAI score [points]							
	*M*	Me	Min	Max	Q_1_	Q_3_	*SD*	*p*‐value
Age group								
21–30	41.2	42.0	30.0	49.0	37.5	45.0	5.2	<.001*
31–40	37.7	38.0	20.0	48.0	32.0	44.0	7.1	
41–50	33.5	34.0	19.0	48.0	29.0	38.0	6.2	
51–60	32.5	32.0	16.0	46.0	28.5	36.5	6.1	
>60	31.2	31.5	22.0	39.0	28.0	36.0	5.9	
Education								
Secondary	32.0	32.0	8.00	47.0	28.0	37.0	6.2	<.001*
Bachelor's degree	35.3	34.0	18.0	48.0	30.0	41.0	6.8	
Master's degree	35.7	36.0	18.0	49.0	31.0	42.0	7.8	
Work position								
Ward nurse	35.6	35.5	20.0	47.0	31.0	41.0	6.6	.42*
Coordinating nurse	32.5	32.0	18.0	45.0	29.0	36.0	6.8	
Unit nurse	33.8	34.0	16.0	49.0	29.0	38.0	7.0	
Other position, e.g. surgical	34.0	34.5	21.0	47.0	29.0	39.0	6.3	
Work in more than one place								
Yes	34.6	35.0	18.0	49.0	29.0	40.0	7.2	.15**
No	33.6	33.0	16.0	49.0	29.0	38.0	6.7	
Hospital ward								
Conservative	34.2	35.0	18.0	49.0	29.0	39.0	6.8	.57**
Surgical	34.0	33.0	16.0	49.0	29.0	38.0	7.0	

Abbreviations: *M*, mean; Max, maximum value; Me, median; Min, minimum value; *N*, number of participants; Q_1_, lower quartile; Q_3_, upper quartile; *SD*, standard deviation.

* Kruskal–Wallis test.

** *U* Mann–Whitney.

The analysis of the relationship between the value of the WAI and the nurses' education level showed that statistically significant relationships exist between secondary education and bachelor's degree and between secondary and graduate education (*p* < .001; Table 2). This means that nurses with higher education scored higher WAI scores than nurses with secondary education.

Another statistically significant dependence showed that with increasing seniority, the ability to work decreases (*r*_s_ = −0.26; *p* < .001). The analysis of the relationship between the WAI and the work position of nurses, work in more than one place, hospital ward did not show statistically significant differences (*p* > .05) (Table 2).

### 3.3 Assessment of the impact of selected variables on work ability

Table 3 contains the impact of selected parameters on work ability (WEI scores). The following variables were included in the analysis: age [years], work seniority [years], age group, education, work position, work in more than one place and hospital ward. Linear regression analysis in a one‐factorial model showed the effect of age (*B* = −0.25, *p* < .001), work seniority (*B* = −0.19, *p* < .001) and education (masters' degree: *B* = 1.41, *p* = .012; ref. secondary) on work ability. The multifactorial model showed a statistically significant impact of seniority on work ability. For people with longer work experience, the ability to work decreases (Table 3).

**TABLE 3:** Single‐factor and multi‐factor linear regression analysis of the impact of selected variables on work ability (WAI score)

	WAI score [points]									
	One‐factor linear regression analysis					Multi‐factor linear regression analysis				
	*B*	*SE*	*t*	*p*‐value	*β*	*B*	*SE*	*t*	*p*‐value	*β*
Age [years]	−0.25	0.04	−6.55	<.001	−0.36	–	–	–	–	–
Work seniority [years]	−0.19	0.04	−4.37	<.001	−0.29	−0.25	0.05	−5.26	<.001	−0.38
Age group										
21–30	Ref.					Not included in the model				
31–40	2.48	1.01	2.45	.015	0.17					
41–50	−1.70	0.68	−2.48	.014	−0.16					
51–60	−2.71	0.72	−3.78	<.001	−0.25					
>60	−4.03	1.61	−2.51	.013	−0.21					
Education										
Secondary	Ref.					–				
Bachelor's degree	0.92	0.55	1.69	.09	0.11	–	–	–	–	–
Master's degree	1.41	0.56	2.52	.012	0.17	–				
Work position										
Ward nurse	Ref.					–				
Coordinating nurse	−1.48	1.18	−1.25	.21	−0.09	–	–	–	–	–
Unit nurse	−0.16	0.65	−0.24	.81	−0.01	–				
Other position, e.g. surgical	0.02	1.01	0.02	.98	0.00	–	–	–	–	–
Work in more than one place										
Yes	Ref.					–				
No	0.46	0.39	1.19	.24	0.06	–	–	–	–	–
Hospital ward										
Conservative	Ref.					Not included in the model				
Surgical	−0.11	0.45	−0.24	.81	−0.01					

Note

Adjusted *R* ^2^ = 0.14.

Abbreviations: *B*, non‐standardized regression coefficient *B*; *SE*, standard error; *t*, *B*/standard error; *β*, standardized regression coefficient *β*.

## 4 DISCUSSION

Poland and many other European countries are struggling with a shortage of medical personnel. This ranges from the ageing of the general population to the premature abandonment of the nursing profession. This makes it all the more important to investigate the factors that directly or indirectly affect the nurses' employment status and ability to work. Little research has been done on Polish nurses' ability to work and the possible causes of their inability to do so.

The observed demographic tendency towards an increase in the proportion of older people (i.e. those aged 60–65 and over) is, of course, not limited to Poland; it is a reality that many other countries face both Europe and worldwide. In Europe, Scandinavian countries – most notably Sweden – exhibit the highest ratios of older people (Bakalarczyk, 2012). In Poland, we can also observe a dynamically increasing percentage of older people. The continuing trend of population ageing will directly impact health, nursing and care services shortly.

Poland is suffering from an acute shortage of nursing staff. This becomes apparent if one compares the number of nurses employed per 1,000 inhabitants in Poland to that of OECD countries. Its rate of 5.2 nurses per 1,000 inhabitants ranks it the bottom of the list (Bakalarczyk, 2012; Organisation for Economic Co‐operation & Development, 2017).

Zając and Szpakowski (2014) report that in 2006 the World Health Organization estimated the health sector's deficit at 4.3 million nurses, doctors and other employees; by 2013, it had risen to 7.2 million. By 2035, it is projected to surpass 13 million. The problem is compounded by the ageing of the nursing staff and the inadequate generation replacement (the average age of nurses in Europe varies between 41 and 45 years). In addition to the serious shortages, the growing absenteeism observed in the nursing profession, which is caused by increasingly frequent sick leaves as a result of work‐related illnesses or occupational stress, further aggravates the situation (Lima et al., 2016).

Our research shows that the average age of nurses is less than 47 years, while the oldest nurses in the study were 65. The average age of nurses participating in the study is four years lower than the average age of the entire population of nurses in Poland in 2016, which was almost 51 years. Moreover, the study showed that the most numerous age groups were 41–50 and 51–60 years – 35% and 28%, respectively. These results demonstrate that the problem of the ageing of the nursing staff also concerns the Polish health care system.

On the other hand, we observed that the age group of nurses under 30 years is less than 10%, which may substantially negatively affect Poland's workforce. Another Polish study by Haczyński et al. (2017) demonstrated that the average age of a Polish nurse in 2008 was 44.19 years, increasing by about six years to 50.1 within the analysed period of 2004–2012. The population of nurses aged above 65 years is over four times bigger than the younger population (21–25 years old). Thus, about 65% of the nurses studied represent a group between 41–60 years of age, and nearly 85% are over 40 years old. Also, Serafin and Czarkowska‐Pączek (2019) observed that only 18.6% of nurses were aged below 29 years in their nationwide cross‐sectional Polish study survey. This trend is also observed in other counties around the world (Halcomb et al., 2018; Spiva et al., 2011).

It should be emphasized that urgent actions are needed to reduce the shortage by making the profession more attractive to young people in Poland. Another important problem described by Szpakowski et al. (2019) to be considered regarding the chronic shortage of health workers and the migration of nurses, which may be crucial in the near future, is that the migration from Poland is not monitored in a reliable way.

Statistical analyses aside, it is self‐evident that ageing diminishes the ability to work. This is a natural consequence of decreased psychophysical capacity, which may manifest in slower reaction times or increased cardiovascular and musculoskeletal diseases (Makowiec‐Dąbrowska et al., 2008). The self‐assessment of one's work ability is the result of the relation between the requirements of work in terms of physical and mental effort and the employee's physical and mental capacities and skills, as well as his or her state of health and judgement of functioning in a particular organisational and social situation. The WAI tool makes it possible to quantitatively present subjective assessments of current and future workloads, physical and mental capabilities to overcome them, and health and its impact on work ability (Makowiec‐Dąbrowska et al., 2008). Szara et al. (2017) and Rostamabadi et al. (2017) emphasize that the efficiency of a nurse's work is a combination of many factors and should thus be viewed in a multidimensional way. Apart from a physical condition, other work efficiency determinants are organisational culture, leadership, planning and evaluation of nursing care, stress levels and social support. Therefore, monitoring workers' employability could help employers diagnose and take corrective action in the work environment, including promoting healthy lifestyles and providing appropriate training (Abbasi et al., 2017; Silva et al., 2016).

Nurses working in inpatient facilities were analysed in light of the factors as mentioned above. The average seniority in the study group was 23.5 ± 9.6 years. It should be noted that the nursing profession's requirements are constant: the workload is the same, regardless of age. The average WAI among the respondents indicates a moderate ability to work, including a four‐point scale: mediocre, moderate, good and excellent. The percentage of moderate work ability is as high as 46.4%. The study showed statistically significant differences in the work ability of nurses working in surgical wards compared to those working in conservative wards. There was also a significant negative correlation between the aptitude to work index on the one hand and age and seniority on the other. This confirms that with age, the body's capacities decrease, which reflects a decrease in the WAI. In the youngest age group (21–30), the average value of the WAI was the highest, amounting to 41 points, which means good ability to work. In the oldest age group (over 60), the average WAI was 30 points, which means moderate ability to work.

In their study on the application of the WAI to people over 50 years of age, Čeledová et al. (2014) compared the WAI results obtained from nurses in various European countries and Israel. They showed that in a study group of Czech nurses and midwives aged between 51 and 62, the WAI score was 36.3 points (moderate ability to work). In Portugal, in a study group of an average age of 34.3, the WAI score was 38.7 points (good ability to work). In Israel, a group aged 41.1 on average scored 41.8 points (good ability to work). Polish nurses score the lowest on the WAI. Estryn‐Behar et al. (2005), Camerino et al. (2006) and Carel et al. (2013) have shown that the WAI score decreases with the age of the worker and that the factor with the most significant impact is the physical workload. The relevant literature emphasizes that people who score low on the WAI are more likely to leave the labour market sooner than those who score high. Significant independent factors causing premature total loss of the ability to work (pension transition) include high workloads, repetitive activities, uncomfortable postures, long working hours and shift work (Habibi et al., 2012; Makowiec‐Dąbrowska et al., 2008).

Considering the ageing of the occupational group of nurses and their shortage in the health care system, one should expect that they would prolong their employment after reaching retirement. That is not the case; however, Bugajska et al. (2011) and Heyam et al. (2018) stress that the ability to work of older people is adversely affected, among others, by shift work – especially at night, working overtime, exposure to chemical factors or heat, physical strain, working in multiple workplaces, the irregular pace of work and occupational stress. Eliminating these factors from jobs occupied by older people can have a positive impact on maintaining their ability to work. Milosevic et al. (2011) emphasize that nurses' satisfactory ability to work directly impacts their quality of life: the higher their ability to work, the better their physical and mental well‐being and social relations.

### 4.1 Study limitations

The study has several limitations. The study group is highly feminized, which reflects the fact that females dominate the nursing profession. Therefore, it was impossible to compare the ability to work between the two genders. Given the physiological and psychological differences between women and men, such a comparison might reveal differences in work ability. Future studies should ensure that a more representative sample of both sexes is selected. Another limitation may have been the difficulty of completing the questionnaire: some questions may have been hard to understand for some respondents. A more advantageous solution would be to ensure the researcher's participation in the questionnaire completion by each respondent. The WAI seems to be a good source of information on potential limitations to the exercise of daily professional duties.

## 5 IMPLICATIONS FOR NURSING MANAGEMENT

Regarding nursing practice, based on the results of this study, programmes can be developed to create or improve healthy working environments that will help prevent occupational diseases and consequently increase productivity. Such measures can also serve to reduce workload and the ensuing fatigue and to increase employee motivation. This study showed that nurses with shorter work experience have a higher ability to work, and this may be due to the fact the workload was higher for those nurses working longer. The lack of development and implementation of a preventive programme for nurses that would enable them to maintain good physical fitness levels, translating into an increased ability to work, may result in a severe shortage of nursing staff in the near future.

## 6 CONCLUSIONS

The nurses surveyed showed a moderate ability to work, which is related to the ageing of this occupational group. This is a significant challenge for the health care system, given that the largest group of nurses was between 41 and 60 years of age. Also, it was observed that nurses with shorter work experience have a higher ability to work.

## CONFLICT OF INTEREST

No conflict of interest has been declared by the authors.

## AUTHORS CONTRIBUTIONS

ŁR, IW and JR made substantial contributions to conception and design. ŁR was responsible for data collection. IW, PK and AK analysed data. ŁR and IW drafted the manuscript. JR, PK and AK revised manuscript critically for important intellectual content. All authors have participated sufficiently in the publication process to guarantee its content and take full public responsibility for the reported study, including its findings and conclusions. The final version is approved to be published by all authors.

## ETHICAL APPROVAL

The research project was approved by the independent Bioethics Committee of the Wroclaw Medical University (approval no. KB‐240/2018 and KB‐426/2018). The study was performed in accordance with the Declaration of Helsinki of the World Medical Association.
